# Psychological, neuroendocrine and inflammatory stress responses in women after miscarriage or stillbirth: investigating early psychobiological adaptations to potential traumatic events

**DOI:** 10.1007/s00702-025-02927-x

**Published:** 2025-04-29

**Authors:** Luis Gerber, Markus M. Müller, Alexandra Oender, Sophia Urbanczyk, Peter Radermacher, Cosima Brucker, Barbara Stein, Christiane Waller, Nicolas Rohleder

**Affiliations:** 1 General Hospital Nuremberg, Department of Psychosomatic Medicine and Psychotherapy, Paracelsus Medical University, Nuremberg, Germany; 2 Anesthesiological Pathophysiology and Process Engineering, Ulm University Hospital, Ulm, Germany; 3 University Women’s Hospital, Paracelsus Medical University, Nuremberg, Germany; 4 Chair of Health Psychology, Institute of Psychology, Friedrich-Alexander-Universität Erlangen-Nürnberg, Nägelsbachstr. 49a, 91052 Erlangen, Germany

**Keywords:** Miscarriage, Stillbirth, Potential traumatic event, Post-traumatic stress disorder, Trauma, Stress reactivity, Biological response

## Abstract

**Background:**

Miscarriage (MC) and stillbirth (SB) can be considered as potentially traumatic events (PTE) and affect approximately 10–20% of all pregnancies. PTEs can lead to the development of post-traumatic stress disorder (PTSD). While the psychobiology of PTSD is well-understood, our knowledge on psychobiological adaptations shortly after a PTE is limited. This study aimed to shed light on early psychobiological changes associated with MC and SB.

**Methods:**

We included 25 women who had experienced a MC/SB within the previous three months and compared them with 28 healthy control women. All participants were asked to attend a study appointment, during which they participated in a socially evaluated cold-pressor test (SECPT) to induce psychosocial stress. Saliva and blood samples were collected at rest, immediately and at 20, 45 and 90 min after the SECPT. We determined salivary cortisol levels and α-amylase (sAA) activity, and plasma interleukin-6 (IL-6) concentrations. We assessed symptoms of PTSD, anxiety and depression using self-report questionnaires.

**Results:**

Women who had experienced MC or SB reported significantly more symptoms of PTSD (*p* < 0.001) and anxiety (*p* < 0.001), when compared to the control group. Despite elevated psychological distress in the MC/SB group, there were no significant differences of salivary cortisol, sAA and IL-6 levels between the two groups at rest or after SECPT induced stress.

**Conclusions:**

Despite the high psychological strain on women after MC/SB, the stress is not yet reflected at a biological level. These results highlight the complex relationship between early trauma, PTSD symptoms, and biological responses. Further research is needed to understand the long-term effects of trauma related to MC/SB, and the development of PTSD, as well as the underlying mechanisms contributing to the observed psychological and biological changes.

## Introduction

The prevalence of potentially traumatic events (PTE) in the general population is reported to be around 30%, differing slightly between various studies and nationalities, with rates up to 70% in the United States (de Vries and Olff 2009; Maercker et al. 2008, 2018; Kessler et al., 2010). Between 15 and 25% of people experiencing a PTE develop post-traumatic stress disorder (PTSD) (Gill et al. 2009) (White et al. 2015). Men and women experience trauma differently, with women being more vulnerable to high impact traumatic experiences, which may at least partially explain the higher prevalence of PTSD in women (Olff 2017).

Pregnancy and birth are significant life events, which in most cases are expected to bring a positive change into someone’s life. However, every pregnancy also harbors the risk of miscarriage or stillbirth (MC/SB). Approximately 10 to 20% of all pregnancies end in miscarriage (Quenby et al. 2021), and around 1.4% end in stillbirth (Hug et al. 2021). A miscarriage, as well as stillbirth (Abiola et al. 2022), can be considered a potentially traumatic event (PTE). Although there are differences between stillbirth and miscarriage, we will, for the purpose of this article, be looking at both experiences together as potentially traumatic events, without differentiating between the two phenomena. Around 29% of women who had experienced a miscarriage developed symptoms of PTSD one month later and 18% were still experiencing these symptoms nine months later (Farren et al. 2020). Depression and anxiety are also not uncommon, with up to 25% of women reporting either moderate-severe depression or moderate-severe anxiety or both following a miscarriage (Farren et al. 2018, 2020), and similar results were found after stillbirth (Westby et al. 2021) It is known that anxiety and possibly subacute trauma consequences occur in the early phase after miscarriage or stillbirth, comparable to the way other life events are processed (Farren et al. 2020).

Over the past decade, research has increasingly focused on somatic correlates and consequences of PTSD and other stress-related disorders, such as fibromyalgia or irritable bowel syndrome, cardiovascular, or autoimmune diseases (Afari et al. 2014; Gradus et al. 2015; Sumner et al. 2017, 2023). For example, PTSD can lead to a 2.93 times higher risk of functional somatic symptoms, and trauma exposure alone can lead to a 2.7 times higher risk of these functional somatic symptoms (Afari et al. 2014). Patients with PTSD also show higher risk of diabetes than healthy individuals (Vancampfort et al. 2016). Women endorsed more health symptoms than men, but no sex differences were noted in the reported number of conditions (Pacella et al. 2013; Ryder et al. 2018).

Somatic diseases associated with PTSD are mediated, to some extent, by chronic low grade inflammation (Couzin-Frankel 2010; Dantzer 2009; Furman et al. 2019; Rohleder 2019), as measured, for example, by C-reactive protein (CRP; Powers et al. 2019). Dysregulated stress systems may ultimately involve disinhibition of inflammatory processes (Hori et al. 2019; Neigh and Ali 2016; O’Donovan et al. 2015; Pace and Heim 2010; Rohleder and Karl 2006). In their meta-analysis, Passos et al. showed that interleukin-6 (IL-6) levels were almost consistently higher in patients with PTSD compared to healthy individuals (Passos et al. 2015). While studies have found effects of cortisol and inflammatory alterations on the development of PTSD (Breslau 2009; Cohen et al. 2006; Olff and van Zuiden 2017; Walsh et al. 2013), the exact involvement of these systems and its directionality still remain unclear (Sumner et al. 2020).

When trying to understand the mechanisms linking PTSD with health outcomes, the biological stress systems, and their interactions with the inflammatory system have been the subject of research. The two main biological stress axes, i.e., the hypothalamic-pituitary-adrenal (HPA) axis and the autonomic nervous system (ANS), are assumed to play a role in converting social environmental adversities into proinflammatory modifications (Almeida et al. 2021; Rohleder 2019; Slavich and Irwin 2014). Dysregulations of the HPA axis are associated with the development of PTSD and other stress-related psychiatric disorders (Kessler et al. 2010; Raison and Miller 2003). This system, with cortisol as its major end product, is the main coordinator of the neuroendocrine stress response (de Kloet et al. 2005). Contrary to a few noteworthy studies (Klaassens et al. 2012), most literature and meta-analyses are in line with the general notion that PTSD is associated with a higher negative feedback sensitivity (de Kloet et al. 2005) and hypocortisolism. With regard to the second pathway, the ANS, many studies report a consistent over-activation of the sympathetic nervous system (SNS) including the sympathetic adrenal medullary (SAM) system, as assessed via endocrine markers and electrophysiological measures such as heart rate variability (HRV), in association with PTSD (Fonkoue et al. 2019; Pan et al. 2018; Sherin and Nemeroff 2011).

While some knowledge exists regarding the general correlates between stress and PTSD, less is known about the reactivity of these systems in response to acute stress. For example, Roelofs et al. (Roelofs et al. 2009) reported a blunted cortisol response when they compared cortisol reactivity to the Trier Social Stress Test (TSST) of patients with anxiety disorders and PTSD to healthy controls. In contrast, (Zaba et al. 2015) did not find any significant differences in cortisol reactivity. Some studies also revealed differences in the salivary alpha amylase (sAA) reactivity (Kuras et al. 2017; von Majewski et al. 2023) or HRV of PTSD patients (Schneider and Schwerdtfeger 2020). This indicates that a relationship might exist between ANS dysfunction and PTSD symptoms. To our knowledge only few studies investigated differences of acute inflammatory stress reactivity in PTSD patients compared to healthy controls, and found pro-inflammatory response to be unaltered, but anti-inflammatory cytokine concentrations and response were more pronounced in PTSD (Renner et al. 2021, 2022; von Majewski et al. 2023).

There is currently no published study that investigated inflammatory stress reactivity shortly after a potentially traumatic event (PTE), and prior to the potential development of PTSD. This is the main research gap we aim to assess with the present study. The role of inflammation in PTSD is still somewhat unclear and results are not fully consistent (Quinones et al. 2020). There are still many unanswered questions, in particular regarding the influence of inflammation on the development of PTSD symptoms. Preliminary evidence suggests that elevated inflammation may lead to an increased risk of PTSD onset, but, as mentioned before, insight on the causality remains limited (Sumner et al. 2020). As MC/SB may lead to substantial feeling of loss and grief which in turn can lead to symptoms similar to those of PTSD, and can per se also lead to PTSD (Heeke et al. 2017), it is important to acknowledge grief as one of the main driving forces in the development of distress. As we do not differentiate between grief, trauma and PTSD, and do not assess symptoms of grief, we will focus mainly on PTSD symptoms in this paper.

In summary, potentially traumatic events may lead to PTSD and other stress related disorders, but the mechanisms, especially on a biological level, remain unclear. Most studies examined PTSD and its association with the human stress response after PTSD has fully developed and been diagnosed. However, one study examined the relation between cortisol levels in the emergency room and the development of PTSD, concluding that higher cortisol levels were a protective factor (Walsh et al. 2013). So far, to the best of our knowledge, no study has yet investigated whether biological differences emerge in early stages of PTSD, and/or before a diagnosis is made. Thus, we set out to examine stress reactivity in women that had experienced a MC/SB three months before in comparison to a control group without trauma experience. We did consider weeks of pregnancy but for the purpose of this study every loss of a child was weighted the same as traumatic experience highly depends on the interpretation of this loss. According to the literature, we expected women who recently experienced MC/SB to have stronger traumatic symptoms than non-traumatized individuals and more comorbid psychological impairments. We further expected acutely traumatized women to have a lower stress reactivity and higher baseline values for cortisol, sAA and inflammatory markers.

## Methods

### Sample characteristics

The entire sample consisted of *n* = 53 women. We included *n* = 25 women who had experienced MC or SB as a potentially traumatic event in the previous three months. For inclusion, women had to report MC or SB as their main traumatic event on the questionnaire Posttraumatic Stress Diagnostic Scale (PDS; Ehlers et al. 1996) and during Structured Clinical Interview for DSM-V (SCID-5). Since we did not differentiate between those two occurrences, in the following we will use “MC/SB” as a means to summarize both events. Women in the MC/SB group were between 25 and 42 years of age (M = 34, SD = 5). Recruitment was done via different channels: in the maternity wards of several hospitals in Nuremberg, doctors in private practice, midwives, and counseling centers via flyers, posters or life presentations on the study topic. Exclusion criteria were acute addiction, acute suicidal tendency, mental disorders or chronic inflammatory disorders requiring permanent medication, dementia, insufficient knowledge of the German language or inaccessibility of General Hospital Nuremberg (“Klinikum Nürnberg”) from the place of residence (for outpatient therapy sessions). To obtain a comparable sample of healthy controls, *n* = 28 women aged between 18 and 48 years with an average age of M = 27 years (SD = 8) were recruited via notices and advertisements at the Friedrich-Alexander-Universität Erlangen-Nürnberg (FAU) and General Hospital Nuremberg. Exclusion criteria were the same as those of the MC/SB group. However, we excluded women who reported a recent traumatic experience, meaning we excluded women who had experienced any kind of PTE in the last 6 months. We further retrospectively excluded women with a baseline cortisol concentration above 20 nmol/l, as high baseline cortisol levels make a cortisol response unlikely (Janson and Rohleder 2017), resulting in a different final sample size for cortisol, amylase and IL-6, respectively (see below). The study protocol was approved by the Ethics Committee of the Friedrich-Alexander-Universität Erlangen-Nürnberg. The study was registered at the Deutsche Register Klinischer Studien (DRKS); ID DRKS00022989.

### Procedures

The study was conducted in a between-subjects design. Each examination took place in the afternoon starting between 13:00 and 15:00 to minimize the effects of circadian variation of biological parameters, and because cortisol responses to laboratory stress protocols are reported to be more pronounced in the second half of the day (Goodman et al. 2017). At the beginning of each laboratory session, a venous catheter was placed into the forearm for blood collection. Immediately thereafter, a 30-minute resting phase was included to allow participants to recover from a potential stress response due to venipuncture. Following this, baseline blood and saliva collection was conducted. Fifteen minutes later, the Socially Evaluated Cold Pressor Test (SECPT; described below) was administered followed by additional blood and saliva samples at 0 (T0), 20 (T20), 45 (T45) (only saliva) and 90 (T90) minutes post SECPT. Baseline and T90 blood samples were drawn into EDTA tubes (Sarstedt, Nümbrecht, Germany), stored on ice for a maximum of 10 min, and then centrifuged and aliquoted as described below. Saliva was sampled using Salivette collection devices (Sarstedt). Participants were instructed to keep the salivette in their mouth for at least one minute and to move it back and forth, but not to chew on it. After the last sample, healthy controls were debriefed and discharged. Patients were not fully debriefed at this time point, as another arm of the study required them to repeat the same assessment three months later. In dedicated intervals between the blood and saliva sampling, self-report questionnaires were obtained.

For acute stress induction, we subjected all participants to the standardized laboratory stress protocol SECPT (Schwabe et al. 2008). This protocol provides a combination of somatic and psychosocial stressors. Participants were asked to immerse their non-dominant hand including their wrist into ice cold water (0–4 °C) for a maximum of three minutes. The participants were allowed to pull the hand out earlier, but were encouraged not to do so. The participants were carefully observed by an experimenter during the entire test. The standardization of the observer’s behavior was ensured by an exact script to be used in testing of all participants as well as by avoidance of any friendly facial expression. In contrast to the standard protocol, we did not use camera feedback in our SECPT, as the patients were often highly affected by their traumatic event, and we aimed to avoid overburdening them.

### Self-report data

We used the German version of the Posttraumatic Stress Diagnostic Scale (PDS; Ehlers et al. 1996), which specifically targets traumatic experiences, PTSD symptoms with their expression according to DSM-IV, as well as a resulting impairment in different areas of life. In total, the questionnaire consists of 49 items. By means of this procedure, the frequency of PTSD symptoms within the last month can be determined against the background of a traumatic experience with a four-point answer scale from 1 = “not at all or only once a month” up to 4 = “5 times or more a week/almost always”. These results were then compared to the Structured Clinical Interview for DSM-V (SCID-5) to assess potential outliers.

The Impact of Event Scale (IES-R) by (Maercker and Schützwohl 2003) was used to assess the frequency with which respondents experienced intrusive thoughts and avoidant behaviors throughout the past week. 22 items are rated on a 5-point scale ranging from 0 (“not at all”) to 4 (“extremely”). The Psychotherapy Basic Documentation (Psy-BaDo; Heuft et al. 1998) was used to record sociodemographic characteristics and medical history, followed by information on psychological and somatic pretreatments as well as a screening on mental health issues like anxiety, depression, eating habits, sleep and others. The Childhood Trauma Questionnaire (CTQ; Bernstein et al. 1998) is the most widely used screening instrument to assess maltreatment during childhood and adolescence (up to the age of 18). It comprises 28 items divided into five subscales: Emotional Abuse, Physical Abuse, Sexual Abuse, Emotional Neglect, and Physical Neglect. Each item is rated on a 5-point Likert scale ranging from “Never True” to “Very Often True.” The CTQ provides both subscale scores and a total score, allowing for the quantification of the severity of trauma experiences.

To assess social support, we used the “Fragebogen zur sozialen Unterstützung” (F-SozU; Fydrich et al. 2009), which operationalizes social support as perceived or anticipated support from the social environment. The Experience in Close Relationships-Revised (ECR-R; Ehrenthal et al. 2009) is a measurement of adult attachment style. The ECR-R measures the individuals’ attachment based on two subscales, i.e., “Avoidance” and “Anxiety”. In general, avoidant individuals find discomfort with intimacy and seek independence, whereas anxious individuals tend to fear rejection and abandonment.

The State-Trait Anxiety Inventory (STAI) by (Laux and Spielberger 1981) is a measurement procedure for assessing trait anxiety (STAI-T) and state anxiety (STAI-S). For the current study, only the STAI-S was used to assess emotional mood characterized by tension, apprehension, nervousness, inner restlessness, and fear of future events. The German version of the “Mood State Questionnaire (”Mehrdimensionaler Befindlichkeitsfragebogen”; MDBF; Steyer et al. 1997) was used to assess participants’ mood state on three scales: “mood”, “alertness” and “calmness”.

## Biological measures

Salivary alpha-amylase activity and cortisol levels were used as markers for autonomic nervous system reactivity (Rohleder and Nater 2009; Warren et al. 2017), and HPA axis reactivity, respectively. Saliva samples were collected at the time points mentioned above using salivettes. All salivettes were stored at -30 °C until processing. Before measurement, salivettes were centrifuged at 2.000 g for 10 min. Salivary alpha amylase activity was measured with an in-house enzyme kinetic assay using reagents from DiaSys Diagnostic Systems GmbH (Holzheim, Germany), as described for example in (Becker and Rohleder 2020). Salivary cortisol levels were measured using a competitive chemiluminescence immunoassay (CLIA; IBL-International, Hamburg, Germany); to quantify low-grade systemic inflammation, we measured IL-6 concentrations in EDTA blood samples obtained at two time points (see above). Blood samples were centrifuged, and plasma was aliquoted and stored at -80 °C until batch processing at the end of data collection. Interleukin-6 concentrations were determined using a commercially available high-sensitivity ELISA (Quantikine HS; R&D Systems, Minneapolis, MN, USA), with a lower limit of detection of 0.09 pg/ml. Inter- and intra-assay coefficients of all assays were below 10%.

### Statistical analysis

All statistical analyses were performed using SPSS Statistics 29 (IBM Corp., Armonk, NY, USA). Normality of distribution was assessed using the Kolmogorov-Smirnov test. Due to violation of normal distribution, biological data (Cortisol, sAA, and IL-6) were transformed by means of the natural logarithm (ln) prior to further statistical analysis. For cortisol, participants with baseline cortisol concentrations higher than 20 nmol/l were excluded from further analysis, as this usually indicates anticipatory stress or stress response to the laboratory environment and/or catheter placement, which resulted in the exclusion of one woman from the MC/SB group and one woman from the control group (Janson and Rohleder 2017). To quantify cortisol and sAA stress reactivity, we computed the area under the curve with respect to ground (AUCg), as well as the AUC with respect to increase (AUCi) (Pruessner et al. 2003). For all parameters, we also calculated the maximum increase as an additional index of stress induced change. To calculate the maximum increase, the baseline level of each parameter was subtracted from the peak level (in the case of IL-6, with only two levels, we computed the difference between the second and the first measurement as “increase”). ANOVAs for repeated measures with the between-subjects factor group (MC/SB vs. controls) and the within-subjects factor time were computed for cortisol, sAA, and IL-6. Greenhouse–Geisser correction was applied where appropriate (indicated by decimal degrees of freedom values). Due to age differences between groups, age was added as a covariate to all AN(C)OVAs. ANOVAS were also used to test for group differences in preliminary analysis. Data is reported as means +/- SEM, and *p* < 0.05 was set as the criterion for significance.

## Results

### Preliminary analysis

As we had different sets of missing data in our analyses of the three biological outcome variables, the final sample sizes differed (sAA: *n* = 24 MC/SB vs. *n* = 25 controls; cortisol: *n* = 23 MC/SB vs. *n* = 26 control; IL-6: *n* = 28 control vs. *n* = 22 MC/SB). Both groups did not differ significantly in prior trauma experiences (CTQ: F[1,48] = 0.20; *p* = 0.66)) and social and relationship support (F-SozU: F[1,51] = 1.14; *p* = 0.29; ECR-RD Anxiety: F[1,49] = 1.32; *p* = 0.26; ECR-RD Avoidance: F[1,49] = 0.02; *p* = 0.89). There was no relationship between PDS subscales or the SCID PTSD scores and the CTQ subscales (not shown). We also computed a variable that included any participant that was in any of the categories of the CTQ higher than the lowest score (meaning any kind of traumatic childhood experience). There was no relationship between this variable and PDS or SCID Scores. (all χ²: p = n.s). The control group had a significantly lower body mass index (BMI; M = 21.5, SD = 2.61) compared to women in the MC/SB group (M = 24.7, SD = 4.93; F[1,50] = 8.98; *p* = 0.004), but BMI showed no correlation with the reactivity of any of the biological markers (all *r* < 0.16; all *p* > 0.10). Both groups differed in age, with the control group being younger (M = 27 years, SD = 7.41) compared to women after MC/SB (M = 34 years; SD = 4.61; F[1,51] = 15.59, *p* < 0.01). As age was significantly related with reactivity scales (Table 1), we decided to control for age in all further analyses of these biomarkers.

**Table 1:** Associations of age and BMI with stress reactivity

	Cortisol			Amylase (sAA)			IL-6
Measure of stress reactivity	AUCg	AUCi	max. increase	AUCg	AUCi	max. increase	increase
Age	-0.29* (0.037)	0.46** (0.001)	0.37** (0.008)	-0.01 (0.95)	-0.13 (0.90)	-0.09 (0.55)	-0.34* (0.017)
BMI	-0.08 (0.61)	0.15 (0.29)	0.16 (0.27)	-0.04 (0.79)	0.01 (0.95)	0.00 (0.99)	-0.02 (0.91)

Pearson correlations reporting r (p-level)

### Psychological measurements

Results of self-report scales are shown in Table 2. As main outcomes to assess traumatization, PDS and IES-R were used. Women after MC/SB showed significantly more trauma symptoms (PDS *F*[1,47 ] = 75.85 *p* < 0.001) than their control counterparts. The different subscales for PDS (PDS Intrusion: *F*[1,48] = 62.86, *p* < 0.001; PDS Avoidance: *F*[1,48] = 71.50, *p* < 0.001; PDS Hyperarousal: *F*[1,47] = 20.93, *p* < 0.001), and IES-R (IES-R Intrusion: *F*[1,36] = 37.65, *p* < 0.001; IES-R Avoidance: *F*[1,37] = 13.14, *p* < 0.001; IES-R Hyperarousal: F[1,37] = 21.18, *p* < 0.001) showed similar results. Furthermore, baseline state anxiety in the MC/SB group was significantly higher than in the control group (*F*[1,50] = 18.36, *p* < 0.001). Similar results were found for the MDBF scales, with lower mood *F*[1,50] = 49.79, *p* < 0.001), alertness (*F*[1,50] = 24.64, *p* < 0.001), and calmness (*F*[1,50] = 12.05, *p* < 0.001) at baseline in the MC/SB group.

With regard to stress-induced changes in anxiety and mood, repeated measures ANOVA did not reveal any significant time effects or group by time interactions for STAI (time effect: *F* [1,50] = 0.03, *p* = 0.86; interaction: *F* [1,50] = 0.24, *p* = 0.62). While mood decreased significantly over time in all groups (*F*[1,48] = 8.73, *p* = 0.005), there were no changes in alertness (*F*[1,48] = 0.11, *p* = 0.74) and calmness (*F*[1,49] = 0.44, *p* = 0.51), and there were no differences in stress-induced mood changes between the groups (group by time interactions; mood: *F*[1,48] = 0.04, *p* = 0.94; alertness: *F*[1,48] = 1.87, *p* = 0.18; calmness: (*F*[1,49] = 0.82, *p* = 0.37).

**Table 2:** Results of self-report scales reporting mean (SD)

Measure		Control Group		MC/SB	
SCID	PTSD	n/a		8 women displayed symptoms of clinical PTSD	
	Depression	n/a		16 women displayed symptoms of clinical depression	
PDS	overall	3.75 (6.10)		19.00 (6.15)	
	Intrusion	0,88 (2.10)		5.92 (2.38)	
	Avoidance	1.32 (2.50)		8.28 (3.28)	
	Hyperarousal	1.54 (2.36)		4.80 (2.61)	
IES-R	Intrusion	7,62 (6,9)		21.00 (6.11)	
	Avoidance	4,86 (6.25)		14.44 (8.69)	
	Hyperarousal	3,57 (6.32)		13.52 (6.56)	
*Stress-induced changes in affect*		*Pre*	*Post*	*Pre*	*Post*
STAI-S	State Anxiety	35.67 (6.31)	35.46 (7.62)	44.20 (8.01)	44.84 (7.38)
MDBF	Mood	17.67 (1.94)	16.65 (2.58)	13.36 (2.45)	12.40 (2.91)
	Alertness	15.93 (2.92)	15.15 (3.76)	11.24 (3.85)	11.80 (3.23)
	Calmness	16.44 (2.42)	15.59 (3.03)	13.24 (4.08)	13.36 (2.74)

### Biological stress reactivity

**Fig. 1:**
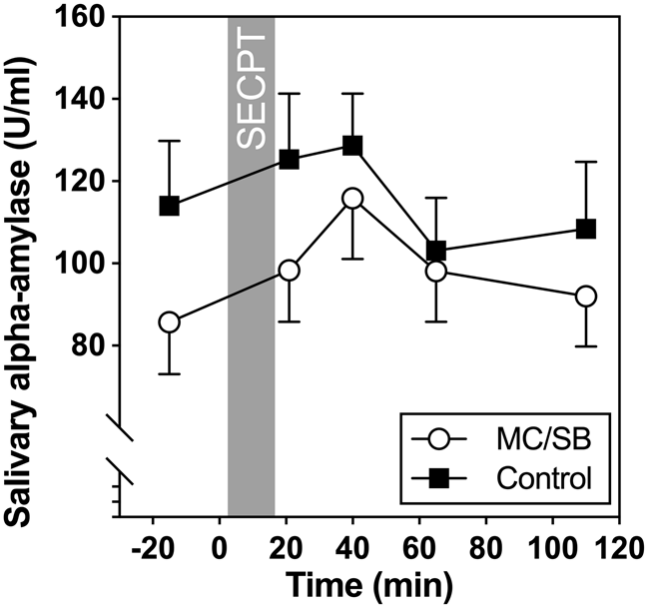
Salivary alpha-amylase activity in the two groups before and after SECPT

As shown in Fig. 1, salivary α-amylase activity did not significantly increase in either group (*F*[2.74,125.84] = 1.94, *p* = 0.13). In addition, neither a significant group effect *F*[1,46] = 1.22, *p* = 0.27), nor a group by time interaction was observed (*F*[2.74,125.84] = 1.70, *p* = 0.17). Groups also did not differ significantly with regard to stress response indices (AUCg: *F*[1,46] = 1.04, *p* = 0.31; AUCi: *F*[1,46] = 2.84; *p* = 0.099; maximum increase: *F*[1,47] = 1.59; *p* = 0.214). At baseline the MC/SB group had lower salivary amylase activities than the control group (marginally significant: *F*[1,46] = 3.95; *p* = 0.05). Neither baseline nor stress responses of sAA were significantly associated with PDS (baseline: *r*= -0.22; *p* = 0.15; maximum increase *r* = 0.24; *p* = 0.11), or CTQ (baseline: *r*=-0.08; *p* = 0.59; maximum increase *r*= -0.02; *p* = 0.92).

**Fig. 2:**
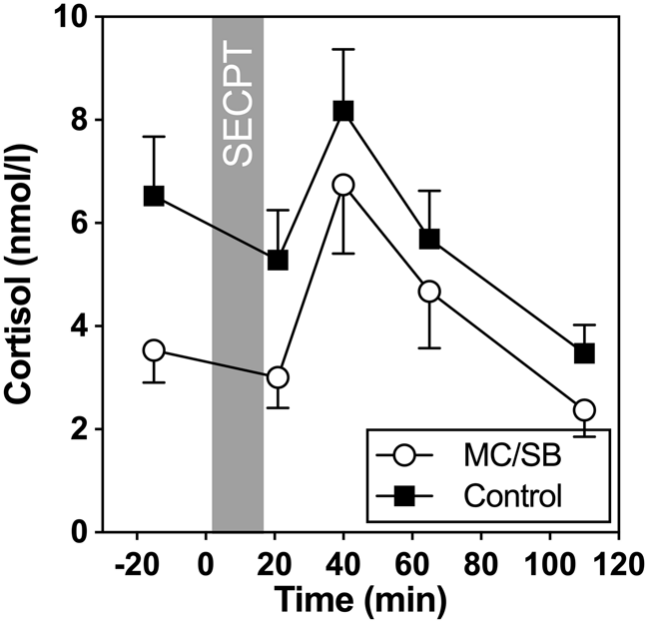
Salivary cortisol concentrations in the two groups before and after SECPT

With regard to cortisol levels, results revealed a significant increase over time in both groups (*F*[1.61,74.02] = 6.47, *p* = 0.005). However, no significant group effect (*F*[1,46] = 1.48, *p* = 0.23), or group by time interaction was found (*F*[1.61,74.02] = 0.47; *p* = 0.58; see Fig. 2). Groups also did not differ significantly with regard to stress response indices (AUCg: *F*[1,46] = 1.54; *p* = 0.22; AUCi: *F*[1,46] = 0.69; *p* = 0.41; maximum increase: *F*[1,46] = 0.64, *p* = 0.43). There were no group differences at baseline (*F*[1,46] = 0.49 *p* = 0.49). Neither baseline nor stress responses of cortisol were significantly associated with PDS (baseline: *r*=-0.24; *p* = 0.11; maximum increase *r* = 0.22; *p* = 0.15), or CTQ (baseline: *r* = 0.0.20; *p* = 0.18; maximum increase *r* = 0.24; *p* = 0.12). Results were similar without exclusion of the two women with high baseline cortisol levels.

**Fig. 3:**
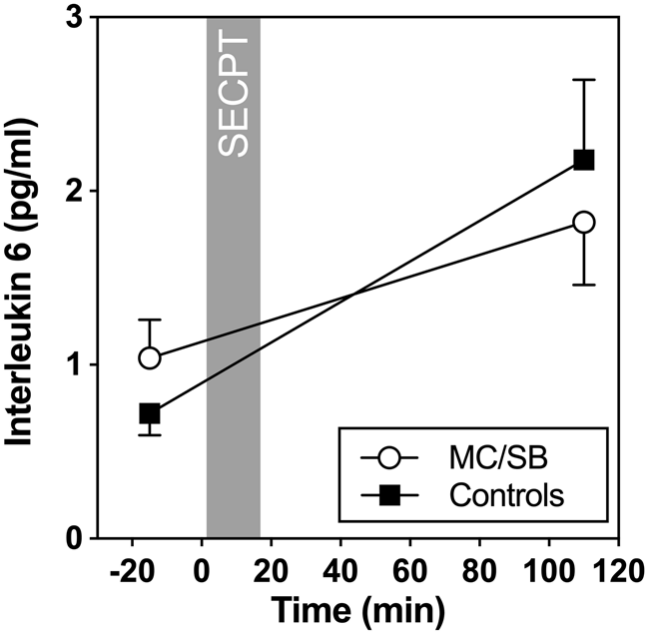
Plasma IL-6 concentrations in the two groups before and after SECPT

With regard to inflammatory reactivity, results showed a significant increase of plasma IL-6 concentrations over time in both groups (*F*[1,47] = 9.1), *p* = 0.004; see Fig. 3). However, no significant group effect was observed (*F*[1,43] = 0.36, *p* = 0.55), and the group by time interaction was not significant either (*F*[1,47] = 0.59, *p* = 0.45). Further, the increase did not differ between groups (*F*[1,47] = 0.59, *p* = 0.45) and there were no group differences at baseline (*F*[1,46] = 1.26 *p* = 0.27). Neither baseline concentrations nor stress response of IL-6 was significantly associated with PDS (baseline: *r* = 0.15; *p* = 0.31; increase: *r* = 0.05; *p* = 0.97) or CTQ (baseline: *r*= -0.06; *p* = 0.72; increase: *r* = 0.10; *p* = 0.52).

### Exploratory analyses

As reported above, we ran correlation analyses between CTQ and baselines as well as response indices of cortisol, sAA and IL-6, to test if women with adverse childhood experiences would show alterations in their regulation of biological stress systems. However, no associations were found (see above). Furthermore, as eight women met the SCID diagnostic criteria for PTSD, and 16 reached those for depression, we also analyzed these subgroups in an exploratory manner to assess differences in reactivity of depressed/non depressed women or women with PTSD/no PTSD and the control group. We only observed differences for sAA activity between the depressed MC/SB and the control group. Women after MC/SB showed lower AUCi *(F*[1,36] = 5.69, *p* = 0.022) and maximum increase *F*[1,37] = 4.10, *p* = 0.050). For AUCg (*F*[1,36] = 1.12, *p* = 0.30), no differences between groups were found. Neither Cortisol nor IL-6 levels showed any differences. No differences were found between PTSD/no PTSD and the biological measurements compared to the control group.

## Discussion

In this study, we set out to investigate the influence of PTE on stress reactivity in women who had experienced a miscarriage or stillbirth (MC/SB), as compared to a healthy control group without PTE. To the best of our knowledge, this is the first study that assessed PTSD symptoms and stress reactivity in the time span up to three months after a PTE had occurred. since not all traumatic events lead to the development of PTSD, our study is unique in that we address potential psychobiological changes, at baseline and in response to acute stress, earlier than other published studies that usually include patients already diagnosed with PTSD.

Our results show that potentially traumatized women showed significantly more self-reported PTSD symptoms, as well as more symptoms of anxiety and depression when compared to women who did not experience a PTE. The symptom severity was lower than the threshold for women with PTSD diagnosis (Foa et al. 2016). Exposure to a laboratory stress protocol induced a significant HPA axis response, as measured by salivary cortisol, as well as an inflammatory response assessed by plasma IL-6, but no significant sAA response. In contrast to our hypotheses, women who had experienced MC/SB did not show significantly different stress responses than control women. However, baseline activities of amylase, but not IL-6 or cortisol levels were marginally significantly higher in women who had experienced a PTE.

Differences in HPA axis function are well documented in PTSD patients (Mason et al. 1986; Yehuda et al. 1990). We did not, however, find any significant differences in cortisol responses between the two groups. (Zaba et al. 2015) compared the cortisol response in a cohort of all female PTSD patients and did find a blunted cortisol response, which appears to be the overall tendency in laboratory stress studies. One of the most recent studies by (von Majewski et al. 2023) comparing PTSD patients with healthy controls did not find a blunted cortisol response either, but did find differences in baseline levels of cortisol between the groups. (Roelofs et al. 2009) reported similar results. They compared patients with PTSD and with social anxiety disorder and a healthy control group, and were not able to find a blunted cortisol response. In these previous studies, an acute stress response was elicited by exposure to the TSST protocol, while in our study, we used the SECPT. This might have resulted in a different stress experience, which could be the reason for not finding significant differences. On the other hand, a key difference between these results and our study is that our traumatized participants had not (yet) developed PTSD. The shorter time period between the potentially traumatizing event and inclusion in our study might therefore not have left enough time for a blunted cortisol response to develop. Such a blunted cortisol response might also be part of a dysfunctional response to the stressor of MC/SB, which would be difficult to detect in a cross-sectional study.

Considering that most studies assessing inflammation in PTSD patients found differences between PTSD patients and healthy controls (Passos et al. 2015; Renna et al. 2018; Rohleder 2014), we expected similar results in our study. However, our groups did not differ in IL-6 baseline levels, nor in IL-6 reactivity, the latter of which is in line with Renner et al. (2021) and von Majewski et al. (2023): Similar to our study, these studies did not reveal differences in IL-6 reactivity either Renner et al. (2021) did, however, find differences in stress reactivity of the anti-inflammatory cytokine IL-10, and, therefore they postulated that acute stress might influence anti-inflammatory cytokines such as IL-10 more than the pro-inflammatory IL-6. This underlying mechanism might have been present in our study as well, but would have remained undetected as we did not measure IL-10. Again, contrary to other studies, we investigated early onset changes shortly after MC/SB. Alterations in inflammation or inflammatory stress reactivity might need more time to develop. (Pace et al. 2006) found increased inflammatory responses regarding IL-6 to psychosocial stress (TSST) in depressed patients with early life stress, but no relationship between childhood trauma questionnaire scores. As such, changes in inflammation might also be partly related to comorbidities with trauma instead of being a direct consequence of trauma. Our findings support the notion that early after a trauma occurs, traumatized individuals do not (yet) show any meaningful changes in baseline systemic inflammation or inflammatory stress responses.

With regard to salivary alpha-amylase, Yoon and Weierich (2016) found higher sAA activity in traumatized patients in response to trauma reminders and von Majewski et al. (2023) reported a diminished sAA response to stress in PTSD patients. These findings were not replicated in our study, as we only found a non-significant trend towards a slightly higher sAA baseline values in the control group, but no differences in sAA reactivity. This is in line with other studies using the SECPT to trigger a stress response (Becker et al. 2019). This result still is surprising, as a number of other studies that used the CPT without social evaluation, did find a significant sAA activity (Skoluda et al. 2015; van Stegeren et al. 2008). This lack of reactivity in our study might have also been the cause of the lack of sAA differences in general in our study. We did however find differences in sAA reactivity when exploratively comparing depressed women after MC/SB to our control group. If women developed a depression after MC/SB they seemed to present with lower reactivity to stressors compared to a healthy control. Depression might be a main driving force of PTSD differences in stress reactivity.

In summary, in contrast to most of the available literature, while we did find stronger PTSD symptoms in women after MC/SB, we did not observe a dysregulated stress axis at that point in time. There may be a variety of reasons for this result. Firstly, it might have been too early in the process of potential PTSD development to observe a chronically dysregulated stress axis. The symptoms we measured occurred in a timespan between three and six months after a potentially traumatic event, and thus might not have fully translated onto a stress axis difference. Secondly, as mentioned before, (Zaba et al. 2015) suggested that there may be two different types of HPA axis reactivity subgroups in PTSD, of which only one would show a blunted HPA axis response. Depending on the diversification of our subgroup this might have had a significant impact on our sample.Using calculations shown in Miller et al. (2013) they speculated that the HPA axis might not directly influence symptoms belonging to the PTSD core symptoms, instead the HPA-axis might interact with trauma-associated dissociative symptoms. Also, as we assessed a sample of women with PTE experience, but without established PTSD, those associated symptoms might not have been present yet.

Our results show that, while a psychological impairment might occur within the first three months after PTE, it does not necessarily translate into sustained biological changes at this rather early time. This implies that an early assessment of the psychological strain after PTE might be very useful in devising a plan to meet PTSD development. This study is further evidence to the consensus that there might be a cascade of psychological and biological reactions that shape the development of PTSD depending on the individual resilience. As such, early interventions might be useful and necessary to counteract PTSD development. Furthermore, the study shows how impactful a MC/SB can be to women and shows the necessity to assess this common occurrence and offer proper help and assistance to women that do experience a MC/SB especially in the early stages of grief.

The following limitations should be considered when interpreting our results. Firstly, we only assessed IL-6 levels at two time points. There might have been more information and maybe even differences if we had been able to assess this reaction for longer. Secondly, our sample size was relatively small, and the two groups did not have the exact same age. The small sample size might also be the reason for descriptive baseline differences we see in all three biomarkers do not reach statistical significance. Our sample was entirely female by nature, and the potentially traumatic event was specifically limited to miscarriage or stillbirth, which makes generalization of the results to other PTE difficult. We did not differentiate between traumatic symptoms and grief symptoms, as in those early stages the symptoms are highly similar (Heeke et al. 2017). The grief process might, however, explain some of the differences in the adaption to the new reality. As our control group comprised women that did not have a recent birth experience and some of them did not even experience a pregnancy yet, it is important to mention the fact that pregnant women may substantially differ from other women and that even uncomplicated pregnancy and delivery can lead to traumatic symptoms and may even be considered in itself a PTE (Horsch et al. 2024). As such, a comparison between women that did experience MC/SB and women that experienced normal birth needs to be made in future studies, and, moreover, future studies need to differentiate between SB and MC as well as to assess other variables such as the number of children the women already have. Further, it might have been advantageous to use the Trier Social Stress Test (TSST) as a laboratory stress protocol for better comparability with other studies. However, our decision in this study is to reduce any potential psychological burden for our participants who had just experienced a significant loss. With regard to assessment of the inflammatory response, it would have been advantageous to obtain more than two samples to better cover the temporal trajectory of the IL-6 response. In line with the previous argument, we decided to minimize sample collection time points and focused on the earliest possible time point of the established plasma IL-6 response to acute stress. Finally, it would have been advantageous to assess the development of the PTSD symptoms in a longitudinal study to determine whether the lack of differences persists when examining only those who do develop PTSD, and vice versa.

In summary, this study highlights the immediate psychological impact of miscarriage and stillbirth, but also suggests that significant biological changes may take more time to develop. Early assessment and support remain crucial to mitigate PTSD risk and assist women dealing with the emotional challenges of MC/SB. However, the study’s limitations, such as a small sample size and exclusive focus on women, should be considered. In the future, longitudinal research with larger and more diverse samples could provide a deeper understanding of the trajectory of trauma-related biological responses and the long-term effects on mental health.
